# Reconstructing patient level survival data from published Kaplan-Meier curves

**DOI:** 10.1016/j.conctc.2025.101542

**Published:** 2025-08-20

**Authors:** Jaromme Kim, Prabhakar Chalise, Jianghua He

**Affiliations:** Department of Biostatistics and Data Science, University of Kansas Medical Center, 3901 Rainbow Blvd, Kansas City, KS, 66160, USA

**Keywords:** Survival analysis, Randomized clinical trials, Time-to-event outcomes, Cancer research, Meta analysis

## Abstract

**Introduction:**

Individual-level patient data (IPD) are helpful for designing clinical trials, conducting meta-analyses, or methodology research. However, such patient level data are not readily available. Multiple methods have been developed for reconstructing survival data using published Kaplan-Meier (KM) survival curves. There has been no practical guidance on an optimal approach or extensive evaluation of the performance of the approach.

**Methods:**

We reviewed several methods of extracting the coordinates of KM survival curves and reconstructing individual-level survival data. Then, we reproduced data from 46 published KM curves. The accuracy of reconstructed data is quantified by comparing hazard ratios (HRs) and their confidence intervals (CIs) estimated from the reproduced data with those reported in the original papers.

**Results:**

The comparison showed a high degree of similarity between the reproduced and original HRs and CIs. In most cases, the differences were less than 5 %. The mean and median absolute percentage differences of 58 reconstructed HRs were 2.85 % and 2.14 %, respectively. These results suggest the reconstruction method reliably reconstructs survival data from KM survival curves.

**Conclusions:**

Based on an extensive number of reconstructions, we demonstrated that reconstructed data provided similar estimates overall to those from published papers. The quality of the reproduced data depends on the presence of noise in the published curves and whether the preprocessing step is properly done.

## Highlights

• Use a semi-automated or automated software program to extract coordinates from KM survival curves to reduce noise and errors.
• Draw two guidelines parallel to the time axis or survival probability axis to specify axis information to the suggested extraction software program.
• According to fifty-eight reconstructed IPD and the corresponding hazard ratio estimates, we have confirmed that the recommended approach for reconstructing IPD reliably generates individual-level survival data.

## 1 Introduction

Randomized clinical trials (RCTs) with time-to-event outcomes usually report estimated hazard ratios (HRs) and their confidence intervals (CIs) based on the Cox proportional hazard (PH) model supplemented by Kaplan-Meier (KM) survival curves. Such published information could be useful for other researchers for designing their own randomized clinical trials, for example, using the estimated HRs for sample size calculations, or conducting meta-analyses. In addition, replication and validation of the observed results from multiple clinical trials are required to generalize the effect of a particular treatment. Therefore, it is helpful to have individual patient data (IPD) to examine and ensure the validity of the results from multiple studies. The aggregate meta-analysis method utilizes only the published results to summarize and present aggregated results, requiring less time and effort than conducting an IPD meta-analysis. For example, some studies gather effect estimates, such as HR, from multiple clinical trials related to anti-programmed cell death protein-1 (PD-1) or anti-programmed death/ligand 1 (PD-L1) [1,2]. These meta-analyses have specific inclusion and exclusion criteria, including whether the study was a randomized clinical trial or whether the trial included specific subgroups of their interest. Notwithstanding that acquiring IPD is laborious, the benefit of IPD meta-analysis can be described as more versatile since sub-group analysis or different analysis methods can be implemented [3]. When researchers want to perform this IPD meta-analysis or secondary analyses on time-to-event outcomes of their interest, individual-level time-to-event data are needed. Unfortunately, original IPD are generally not easily accessible. Privacy protection complicates access to clinical information of human subjects, making it difficult for other researchers to directly obtain the original IPD of a study of interest for further analysis.

Several early studies attempted to obtain hazard ratio estimates from KM survival curves for aggregate meta-analyses [4,5], but those studies were only confined to getting the weighted average of the piecewise HRs not IPD. In addition, for the study design of clinical trials, some researchers proposed methods of pulling quantile information from KM survival curves for sample size calculation [6,7].

Methods have been developed for reconstructing IPD based on published KM survival curves. Generally, two steps are involved to reconstruct time-to-event outcome data: first, getting digitized coordinates from published KM survival curve images, and second, reconstructing individual-level events or censoring time from the extracted coordinates.

In the first step, the digitization step, coordinates from the KM survival curve image can be extracted from public software programs like CurveSnap [8], or ScanIt [9]. The quality of this first step depends on how well users preprocess images or eliminate unwanted regions in the image to improve the software program's performance. After extracting the coordinates of the KM survival curve graph, the second step is the reconstruction step. During this step, the extracted coordinates are processed with an iterative algorithm proposed by Guyot et al. to generate IPD [10]. This algorithm is available for public use but is not user-friendly. Moreover, this method requires users to manually modify the extracted coordinates because they may not follow the non-increasing property of KM survival probability over time due to noises/errors from the coordinate extracting process. An R package, survHE, wraps these R codes into a single R function along with other various features for health economic evaluation [11]. However, this package still does not modify the extracted coordinates to follow the non-increasing trend of KM survival curves. Building on the algorithm outlined by Guyot et al. [10], Liu et al. upgraded the algorithm to reduce rounding errors and remove redundant extracted coordinates [12]. Moreover, they provide a coordinate extraction feature and a coordinate modification feature that automatically adjusts coordinates to maintain the non-increasing trend over time. Liu et al. [12] implemented these features in a user-friendly R Shiny application [13] that enables reproducing IPD along with an R package named IPDfromKM. Users can use the R Shiny application to obtain a small number of IPD relatively quickly. Alternatively, they can generate many datasets of IPD from various studies by calling functions from the IPDfromKM package within loop function codes in R. Recently, a new tool was published that can utilize hue, saturation, and lightness scale of KM curve images to fully automate reconstruction of IPD incorporating k-medoids clustering algorithms [14]. However, the IPD from this fully automated method often shows substantial deviation from the actual KM survival curve, failing to capture the true trend due to various reasons: censoring symbols, dashed line representations, similar color contrasts between curves, or considerably overlapping curves. Zhang et al. [14] suggested that users clean the images carefully before using this tool for the above scenarios. In addition, Guyot's algorithm [10] has another variation in reconstructing IPD of an unreported subgroup using the KM survival curve of the overall cohort and that of the other known subgroup [15].

A significant deviation of reconstructed IPD from the actual IPD could affect the reliability of subsequent analyses. Thus, practical research into reconstruction methods and an evaluation using a large amount of real-world data are needed to confirm the appropriate reconstruction process and the feasibility of the reconstruction method. This paper aims to demonstrate how well users can prepare images of KM survival curves to extract coordinates by showing an example. Then, we aim to evaluate the accuracy of reconstructed IPD generated by the iterative algorithm provided by Liu et al. [12]. We reviewed the method by Liu et al. [12] because it encompasses practical features that other packages lack, such as automatic coordinate modification to maintain the non-increasing trend. Using the results from a large collection of studies, we discussed the application and efficacy of the IPD extraction method in this work.

## 2 Methods

This section first explains how to get IPD, beginning with an image of KM survival curves with an example. The data used in the example are lung cancer data from an R package, survival [16], using a group indicator as sex. As we introduced before, reconstructing IPD consists of two steps: the first step is to digitize KM survival curves obtained from an image of a graph, and the second is to iteratively reconstruct individual-level time-to-event outcomes using the digitized coordinates.

For the digitization step, we need a captured image of KM survival curves, and the time and survival probability coordinates of each group extracted separately. This will be covered in the following Section 2.1. The reconstruction step requires the following information to ensure the accuracy of the reconstructed IPD generated by the algorithm or application [12]: the number of patients at risk at different time points and the corresponding time points as reported in the published paper, which will be explained through Section 2.2. Without such information, the algorithm will still return IPD, but the accuracy of reproduced IPD will not be reliable compared to the actual IPD. Therefore, we include the number of patients at risk at different time points to accurately reproduce IPD.

Then, for the evaluation purpose, we chose 14 immuno-oncology clinical trials which assessed the efficacy of anti-programmed cell death protein-1 (PD-1) or anti-programmed death/ligand 1 (PD-L1) monoclonal antibodies, either as monotherapy or in combination with other agents that were published in peer-reviewed journals or presented at major conferences [[17], [18], [19], [20], [21], [22], [23], [24], [25], [26], [27], [28], [29], [30]]. The papers were selected based on the list provided in a published systematic review on immuno-oncology clinical trials [31]. The list used 63 RCTs to compare various statistical tests with the following exclusion criteria: 1. studies with sample sizes less than 100, 2. primary analysis is not overall survival (OS), or progression-free survival (PFS), 3. no significance level prespecified to PFS, or OS, 4. no public result is available, or 5. studies sponsored by a hospital or a clinical trial cooperative group. Among the list, we chose 14 RCTs that were specifically mentioned by Mukhopadhyay et al. [31], suspecting the proportional hazard assumption, such as delayed treatment effect, crossing hazards between groups, or diminishing treatment effect scenarios. Then, 46 KM survival curve graphs were collected from the 14 published papers including overall, progression-free, intention-to-treat, and other subgroup survival analyses for reconstructing IPD and evaluation purposes. The graphs were excluded if the numbers at risk and estimated HR were not provided in the published papers. Within the chosen 14 RCTs, the follow-up duration ranged from 12 months to 42 months and the sample size for overall survival analyses ranged from 142 to 1217, and for subgroup survival analyses, the sample size ranged from 33 to 223.

The accuracy of reproduced IPD is quantified as the deviation of the reproduced HRs with their CIs from those provided in the referenced papers. The reproduced HR is calculated from a Cox PH model with an intervention group indicator as the only covariate for each KM curve graph.

### 2.1 Digitizing KM survival curves

In this step, we will explain what considerations need to be made to extract more robust and accurate coordinates from an image of KM survival curves. Various software programs are available to extract coordinates from images, but only a few software programs are useful in the survival analysis domain. Thus, we will focus on two digitizing methods that we consider most user-friendly: the R Shiny application by Liu et al. [12] and CurveSnap [8].

**Fig. 1:**
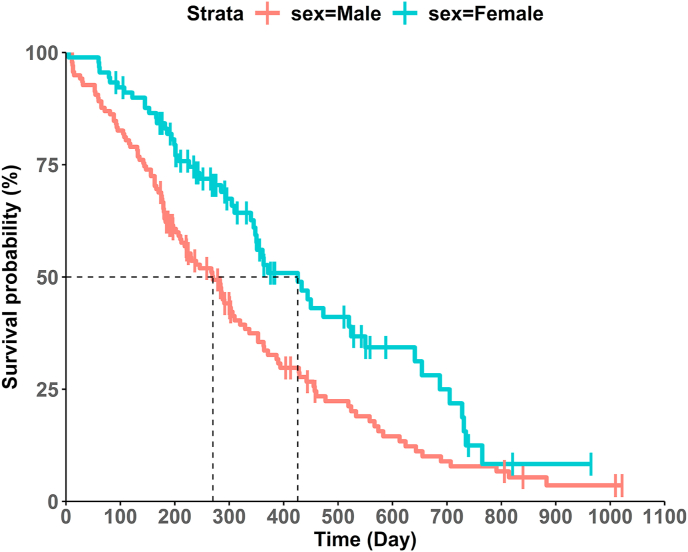
KM survival curves by sex.

Before we move on to how well we can extract coordinates from an image, we should make sure that the captured image contains the time axis, survival probability axis, and KM survival curves, as in Fig. 1 generated by the example lung cancer data. This KM survival curves below are grouped by sex, and the median survival time point is displayed by dashed line for each group.

After obtaining the image of KM survival curves, we need to examine whether the curves overlap significantly or not. If they overlap significantly, which may interfere with the digitizing software program's ability, we recommend providing a separate image for each group to digitize coordinates.

**Fig. 2:**
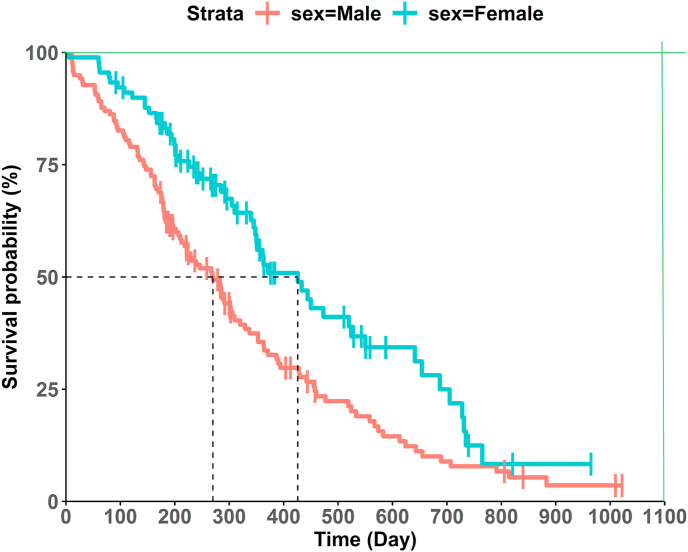
KM survival curves with two added guidelines (green).

Next, we consider whether the KM survival curve graph displays the median or any other quantile survival time on the curves. If the graph displays this information, it would be easier to provide axis information to CurveSnap [8] (when using the R Shiny application, there is no need to consider this. These will be dealt in detail through the following section 2.1.1. and 2.1.2). Otherwise, we recommend drawing two guidelines on the captured image through a basic Paint application, as in Fig. 2. Each guideline is parallel to the horizontal or vertical axis of the original image. These guidelines are helpful in inputting axis information into the digitizing software program CurveSnap [8]. This is recommended when no quantile survival time is displayed on survival curves.

#### 2.1.1 IPDfromKM R Shiny Application

This R Shiny application by Liu et al. [12] is a free website for public use [13]. The website provides features to digitize curves from imported images, reconstruct IPD, etc. Users can use this website to get IPD starting from a captured image of KM survival curves of interest. Consider the KM survival curve with two groups with censoring symbols (vertical marks), as in Fig. 1. After importing the image, users should provide several information to set up axis information according to the instructions on the blue panel from the website (minimum and maximum points for each axis). Then, the users extract coordinates from a KM survival curve for each group separately by clicking the starting point of the curve toward the end of the curve. The users must use as many clicks as possible along the survival curve to extract the trend precisely. The users may find out that extracting coordinates accurately is not easy and that they must pay attention to capture the actual trend of the survival curve. Make sure to click up to the curve's last point to reconstruct the actual IPD well. By clicking along the curve, the users can check their last click on the graph on the bottom left side and undo the last click if it is not accurate. After finishing extracting coordinates, the users could get these coordinate data into a CSV format file.

#### 2.1.2 CurveSnap

CurvSnap [8] is a stand-alone software that can extract coordinates easily from any type of line graph. First, users should import an image of KM survival curves to CurveSnap [8]. Then, users have to set axis information to CurveSnap [8]. It requires users to specify two points that are not on the same horizontal or vertical line. Therefore, it is recommended that users select the origin of the survival curve graph and another point that is easy and accurate to specify. The origin is indicated in most KM survival curve graphs, and another recommended point is if the published KM survival curve visualizes the median survival time (or some quantile survival time) on the curve, then that point would be the recommended point. That is, users need to select the origin and the point on the curve that is expressed as the median survival time (or some quantile survival time) to set the axis information. Otherwise, if there is no quantile survival time displayed on curves, we recommend drawing two guidelines parallel to the time or survival probability axis before users import an image to the software program, as in Fig. 2. These two guidelines are to specify the following points: the origin and the point where the two guidelines intersect.

After setting up the axis information, users can use an eraser tool from CurveSnap [8] to reduce noise coming from censoring symbols (e.g. vertical marks on the curves in Fig. 1, Fig. 2). Then, to extract coordinates, there is a feature that extracts coordinates of the KM survival curve through the curve's connectivity or color. We recommend extracting coordinates by their color, as most of the published KM curves are discerned by different colors. Alternatively, users could extract coordinates by curve connectivity if different shapes, such as dashed and solid lines discern KM survival curves. To get the coordinates of the dashed line, users need to click the dashed segments multiple times along the curve. Users can check the extracted coordinates visually on the right panel and erase any unwanted region extracted by error to get coordinates closer to the actual trend using the erase feature (unwanted regions are not limited to censoring symbols, or axis tick labels). After the extraction, users can get these coordinate data as a spread sheet format file. One thing to consider when using CurveSnap [8] is that it digitizes vertical step down regions of a KM survival curve as a slightly tilted line, which makes the extracted coordinates have innate noise (e.g. the possibility of overestimating number of events). However, the possibility of getting wrong estimates for numbers at risk or number of events seems insignificant, as the extracted coordinates for time have differences less than 0.001.

Many other software programs enable us to get coordinates from images of curves. ScanIt [9] is one of them, providing digitized coordinates from KM survival curves. ScanIt [9] retrieves coordinates by curve connectivity as in CurveSnap [8], but it uses a slightly different method. The software program necessitates at least two points on the curve to be specified, and it returns the coordinates between the two points by the curve's connectivity. Thus, users specify more than two points to enhance the accuracy of the extracted coordinates. This semi-automatic software program works in the presence of censoring symbols. However, there is a proneness of underestimating KM survival probabilities when censoring symbols interrupt the actual trend of KM survival curves. Thus, when using ScanIt [9], removing censoring symbols is recommended to get precise survival probabilities. Due to the conveniences of extraction from CurveSnap [8] and the underestimation trend of ScanIt [9], we recommend using CurveSnap [8] to get coordinates from KM survival curves.

Table 1 provides a comparative summary of the key features of manual, semi-automated, and fully automated digitization methods, which enhances clarity and accessibility for applied statisticians and methodologists (manual method: IPDfromKM R Shiny Application [13], semi-automated method: ScanIT [9], fully-automated method: CurveSnap [8]).

**Table 1:** Features of different digitization methods.

Step & Feature	Manual Digitization: IPDfromKM R Shiny Application [13]	Semi-automated Digitization: ScanIT [9]	Fully Automated Digitization: CurveSnap [8]
**Image Resolution**	High resolution is recommended for accurate clicks	High resolution is recommended for coordinate extraction	High resolution is recommended for automated extraction
**Axis Information**	User clicks minimum and maximum points of each axis	User clicks three independent points (e.g. origin, and two maximum points of two axes)	User clicks two non-aligned points (e.g., origin and a quantile); guidelines may be needed
**Coordinate Extraction**	Click along KM survival curve manually from the starting point of the curve to the end of the curve	Click along KM survival curve (at least two points on curve are needed)	Coordinates extracted automatically based on a selection (connectivity/color); unwanted regions can be erased afterwards
**Output File Format**	csv	txt	CSV, tab- or space- delimited

### 2.2 Reconstructing individual patient data (IPD) iteratively

The iterative algorithm developed by Liu et al. is used to get an IPD from extracted coordinates [12]. Users can use this algorithm through the R Shiny application [13] or R functions (*preprocess()* and *getIPD()*) from the IPDfromKM package in R. When using the R Shiny application, getting a small number of IPD is relatively easy. However, suppose users need to generate many datasets of IPD from various studies. In that case, they can use the IPDfromKM R package within a loop function for faster reconstruction.

Users accessing the R Shiny application can go to the Reconstruct tab to get IPD. Then, they import the extracted coordinates and input the required information, such as the numbers at risk and the corresponding time points to the application. The following information is not required but can be included in the algorithm: the total number of patients and the total number of events. Users can also designate the time interval and the vector of survival probabilities of interest to be used in a secondary analysis report generated automatically by the application.

By clicking a button for calculating IPD, the application iteratively estimates IPD with the extracted coordinates and the input information. Inside the application, it checks whether the first extracted time and the corresponding survival probability are zero and one, respectively. If not, they are modified to have a starting time of zero and a corresponding survival probability of one. The next thing that the application does inside is to find any time point when the extracted survival probability is higher than the extracted survival probability of the immediately preceding time point. Then, the application adjusts the later survival probability to equal the previous one to maintain a non-increasing survival probability function over time. For example, the x (time) and y (survival probability) coordinates for the female group are extracted from Fig. 1 using CurveSnap [8], and saved as in Table 2. The first and second columns represent the raw extracted coordinates (time points and survival probabilities), and the third and fourth columns represent the coordinates after the adjustment. The first row of extracted coordinates (−1.401, 0.998984) is adjusted as (0, 1), followed by increasing time and non-increasing survival probabilities inside the application. This process reduces unwanted noise from extracted coordinates before applying extracted coordinates to the iterative algorithm.

**Table 2:** Extracted time points and survival probabilities before and after adjustment.

Raw Time	Raw Survival Probability	Adjusted Time	Adjusted Survival Probability
−1.401	0.998984	0	1
3.11735	0.995015	3.11735	0.995015
3.87041	0.994519	3.87041	0.994519
4.62347	0.994519	4.62347	0.994519
5.37653	0.994519	5.37653	0.994519
⋮	⋮	⋮	⋮

**Fig. 3:**
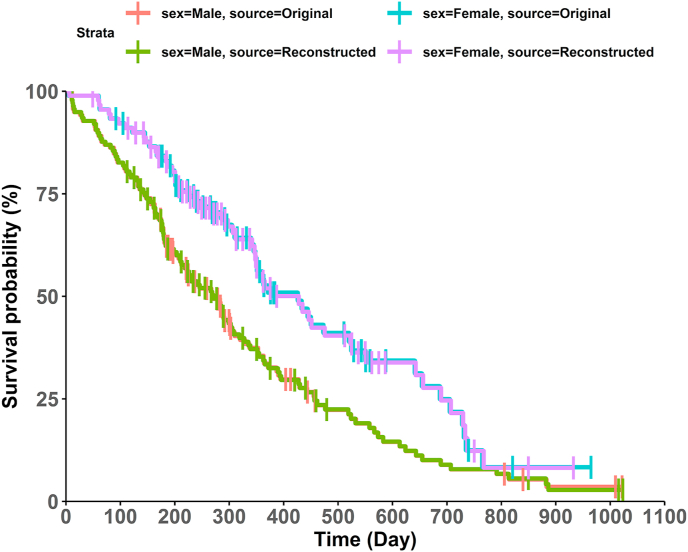
Overlapped reconstructed and actual KM survival curves by sex.

For the lung cancer data example, we generated IPD from the extracted coordinates via the R Shiny application [13] and CurveSnap [8]. Then, KM survival curves by sex were reconstructed, and the original KM survival curves overlapped on the same graph as in Fig. 3. This type of plot can be helpful to see how close the reconstructed IPD is to the actual IPD. We can check whether the reconstructed KM survival curves are close to the actual curves or not.

After reconstructing IPD from the application website, there are four tabs on the right panel of the application website, and the four tabs display Reconstructed IPD, Accuracy assessment, Secondary analysis, and Download report. Users can copy, save or print the reconstructed IPD. The accuracy assessment report includes plots of KM survival probabilities, the number of patients at risk by time, and the difference between estimated survival probabilities and read-in data (Here, read-in data does not mean actual IPD used from the original research. But it only means the extracted and adjusted coordinates as in Table 2). In addition, it shows the root mean square error (RMSE), mean absolute error, maximum absolute error, and the result of Kolmogorov-Smirnov test. Furthermore, users can obtain the secondary analysis reports containing milestone survival time points (as specified by users or default (0.25, 0.5, 0.75)) and their CIs. Those outputs could be saved separately as a comma-separated values (CSV) format file or downloaded as a single report-type file from the download tab for further analysis.

■ Summary of recommendations
  ✓ Capture all parts of KM survival curves, including axis ticks and labels
  ✓ If the KM survival curves do not display median survival time (or some quantile survival time) on the curves, draw two guidelines parallel to the time or survival probability axis to help set up axis information
  ✓ If KM survival curves overlap significantly, generate and import a separate KM survival curve for each group to extract coordinates accurately
  ✓ Although the R Shiny application has all features imbedded in one platform, we found that CurveSnap [8] has the advantages over the application regarding extracting coordinates and the erasing feature that can remove unwanted parts (censoring symbols, tick labels, legends, etc) adaptively from extracted coordinates

## 3 Evaluation

To evaluate the accuracy of reconstructed IPD using the algorithm provided by Liu et al. [12], we generated IPD from 46 KM survival curve graphs sourcing from the 14 immuno-oncology papers which compare the efficacy of anti-programmed cell death protein-1 (PD-1) or anti-programmed death/ligand 1 (PD-L1) monoclonal antibodies, either as monotherapy or in combination with other agents. We used a basic screen capture tool (Windows’ Snipping Tool) to obtain images of the KM survival curves from PDF versions of published articles. During the digitization step, to give axis information and extract coordinates accurately, we drew two guideline lines as in Fig. 2 and generated a separate image for each intervention group. Then, CurveSnap [8] was used for each KM survival graph to get coordinates for each group separately, based on either the color or connectivity of the curve, whichever was most appropriate. Then, coordinate files generated from the same KM survival graph were appended together into one file with a group indicator created.

After creating and preprocessing the digitized coordinates from all the KM survival curve graphs, IPD were reconstructed with both the R functions (*preprocess()* and *getIPD()*) from the IPDfromKM package and a *for()* loop function was used to mass-process the reconstruction process. Then, HRs and their CIs were calculated using the Cox regression model with the group indicator as the only covariate in the model. The estimation of HR was done by using the R package, *survival*, with a *coxph()* function [16]. The reconstructed estimates were compared with those reported in the published papers.

Evaluation metrics were defined to quantify reconstruction performance. The first metric, the percentage of absolute HR difference was calculated as $\frac{\left|HR_{r}-HR_{o}\right|}{HR_{o}}\times 100\;\left(\%\right)$, where $HR_{r}$ represents the reproduced hazard ratio, and $HR_{o}$ represents the original hazard ratio from the published papers. When this metric is close to 0 (%), for example <5 (%), the reconstructed IPD is close to the actual IPD. On the other hand, when this is further away from 0 (%), then the reconstructed IPD deviates from the actual IPD. The second metric, the percentage of non-overlapping region, is calculated as $\left(1-\frac{overlapping\;region}{range\;of\;CI_{o}}\right)\times 100\left(\%\right)$, where $overlapping\;region=\max\left(0,\min\left(U_{o},U_{r}\right)-\max\left(L_{o},L_{r}\right)\right)$, $range\;of\;CI_{o}=U_{o}-L_{o}$, and $U_{i}$, $L_{i}$ with i=o,r representing original and reproduced upper and lower confidence intervals of ${HR}_{o}$ or ${HR}_{r}$, respectively. If this value of the non-overlapping region is low, for example <5 (%), the reconstructed CI overlaps mostly with the range of the published CI so that we can think of the reconstructed IPD as similar to the actual IPD.

## 4 Results

**Fig. 4:**
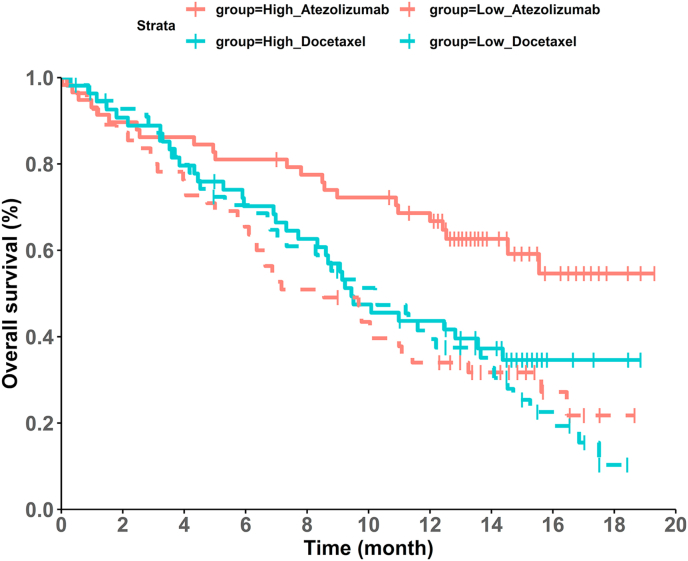
KM survival curves by gene expression and treatment (Atezolizumab and Docetaxel).

Based on the 46 published KM graphs, each KM curve graph was digitized and reconstructed into IPD one at a time using the steps outlined in the Evaluation Section. As some graphs had more than two intervention arms, a total of 58 HRs and their CIs were estimated based on the 46 KM graphs. The mean (standard deviation (SD)) of the percentages of absolute HR difference were 2.85 % (3.42 %), and the median was 2.14 % with the range (0.00–23.25 %). Forty-nine out of 58 HRs (84.48 %) had the percentage of absolute HR difference less than 5 %, 8 cases out of 58 (13.79 %) had the percentage of absolute HR difference 5 % or greater and less than 10 %. The only case with a percentage greater than 10 % was 23.25 %, due to the technical difficulty of digitizing four overlapping KM curves (distinguished by colors and line types) on a single graph. The mean (SD) of the percentages of non-overlapping region for CIs is 4.35 % (4.43 %), and the median was 2.45 % with a range of (0.00–17.78 %). Thirty-five out of 58 (60.34 %) reconstructed CIs had the percentage of non-overlapping region less than 5 %; 16 (27.59 %) reconstructed CIs had the percentage of non-overlapping region of 5 % or greater and less than 10 %; the rest seven cases (12.07 %) were between 10 and 20 %. Overall, the reconstructed HRs and CIs well aligned with the original published values. A full list of reconstructed and original HRs and their CIs is tabulated in Supplementary Table 1. Based on the absolute percentage difference in HRs, the most accurate reconstruction was observed from the comparison between Durvalumab and chemotherapy reported by Rizvi et al. [27], where both the original and reconstructed HRs were identical (HR = 0.93), and the percentage of non-overlapping regions for the CIs was also low (0.71 %). Each group consisted of 209 and 185 patients, respectively and the follow-up length was 36 months. In contrast, the largest discrepancy was observed in the comparison of Atezolizumab versus Docetaxel in the T-effector and interferon-γ high subgroup by Fehrenbacher et al. [18], with an absolute percentage difference in HR of 23.25 % and a percentage of non-overlapping regions for CIs of 13.58 %. The sample size for each group was 58 and 54, respectively and the follow-up length was 20 months. The reconstructed KM survival curves are shown in Fig. 4 to demonstrate the difficulty in digitizing the curves accurately from this case.

**Fig. 5:**
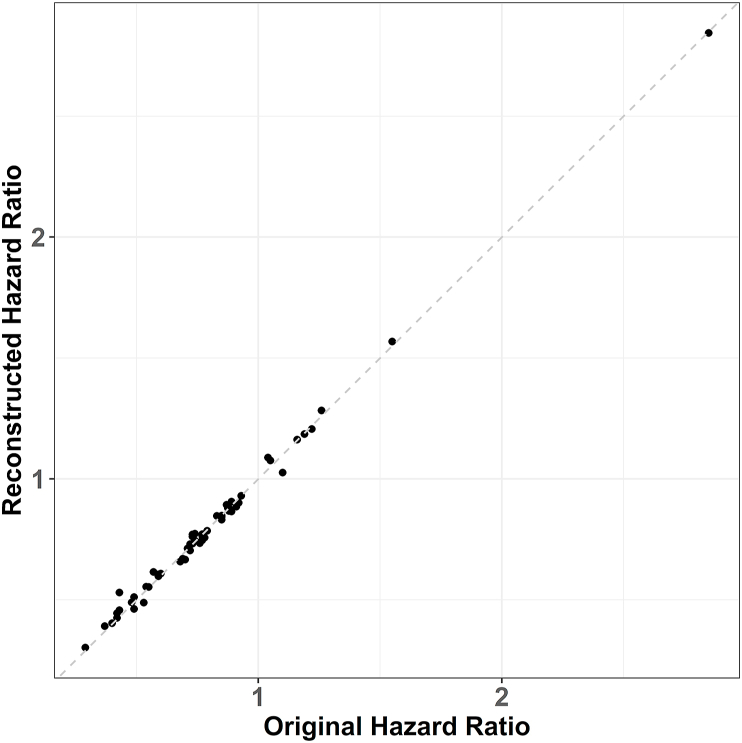
Scatter plot of reconstructed and original hazard ratios (HRs).

**Fig. 6:**
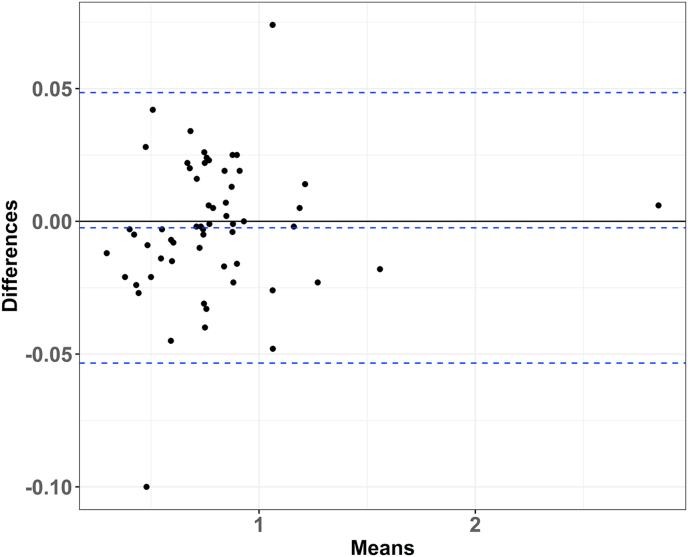
Bland-Altman plot regarding reconstructed and original hazard ratios.

The scatter plot and Bland-Altman plot show visualized comparisons of all reconstructed and original HRs. Scatter plot (Fig. 5) shows that most points align on the diagonal line, indicating a high consistency between the reconstructed and original HRs across the chosen KM survival graphs. Each point in the Bland-Altman plot (Fig. 6) is displayed by the difference in HR between reconstructed and original IPD against the mean value between the reconstructed and original HRs. Most points fall within the limits of agreement, with the exception of two outliers. Those two cases were from the gene expression subgroup analysis by Fehrenbacher et al. [18], the result of which were based on Fig. 4.

**Fig. 7:**
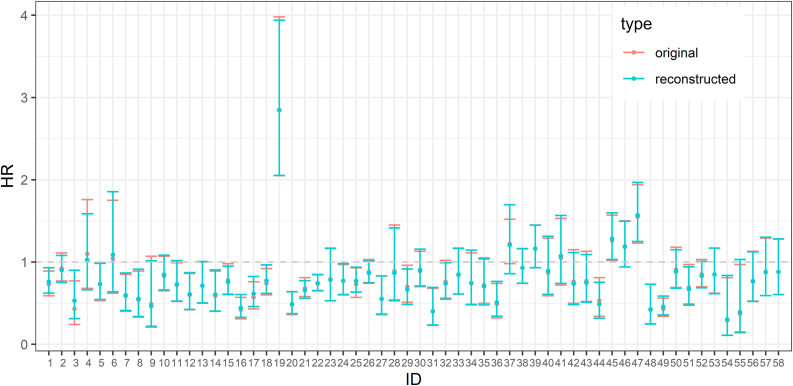
Estimated HRs and their CIs based on the original IPD (red) and the reconstructed IPD (turquoise).

Fig. 7 visualizes estimated HRs, and their CIs based on reconstructed vs. original data. These results suggest that the reconstruction method is overall reliable for reconstructing IPD from most KM survival graphs.

## 5 Discussions

In this study, we reviewed several methods available for reconstructing IPD based on an image of KM survival graph usually provided in the published manuscript for an RCT with time-to-event outcomes and demonstrated the performance of the recommended reconstruction methods.

The digitization step may require a lot of labor as users need to manually erase censoring symbols and overlapping regions properly. Thus, users are tempted to digitize curves directly using the R Shiny application. However, this digitizing step is worthwhile for reconstructing IPD accurately. We recommend using the digitizing software program that provides semi-automated or automated extraction to reduce noise and errors. As mentioned in the Method Section, CurveSnap [8] returns extracted coordinates to have innate noise as the extracted coordinates are slightly tilted for vertical step-down regions of a KM survival curve. This may be a problem when there is a KM survival curve with extreme slope regions. The extreme slope regions refer to a KM survival curve that shows near-vertical declines, rather than the typical stepwise decreases seen in standard KM curves. Such patterns can occur when a large number of events happen within a short time interval and this trend persists over a considerable portion of the follow-up. Upon review of the 46 KM survival curve graphs included in our analysis, we found no instances of curves exhibiting nearly vertical drops; all displayed the usual pattern in survival probability that may not affect the quality of digitization considerably. In our experience and based on a review of the published literature, such extreme vertical declines are rare in real-world clinical trial data. However, if researchers do encounter KM curves with regions of extreme slope or near-vertical declines, users can use ScanIT [9] for digitization, as it can accurately capture vertical segments by assigning identical time coordinates to those points. This helps preserve the true structure of such challenging regions and improve the fidelity of subsequent IPD reconstruction (however, note that ScanIT [9] needs at least two points specified to extract coordinates, requiring more manual specification in the digitization step). Moreover, this innate noise is so slight that it has less impact on estimating numbers at risk and survival probabilities, making CurveSnap [8] our recommended software program.

With respect to image resolution of captured KM survival curve graphs, the use of the Snipping Tool was intended to demonstrate a practical and commonly used approach for researchers seeking to reconstruct IPD from published figures. We acknowledge that lower resolution images may compromise the accuracy of digitized coordinates. Thus, we recommend obtaining the high resolution images available from original or official sources whenever possible.

At the reconstruction step, users should keep in mind that preprocessing the extracted coordinates is an important step as it will ensure that the non-increasing property of KM survival curves and the accuracy of reconstructed IPD. If users do not go through this process, the iterative algorithm may fail to converge or result in inaccurate estimation of the numbers at risk. Both issues can introduce bias, such as misestimating event times or number of events, and ultimately impact the reliability of reconstructed HRs and CIs. To empirically assess the impact, we reconstructed IPD for 15 KM survival curve graphs without this preprocessing step. When the preprocessing step was omitted, the mean (SD) absolute percentage difference in HRs was 6.15 % (8.74 %), and the mean (SD) percentage of non-overlapping regions was 8.11 % (7.21 %), both higher than when preprocessing was applied (The mean (SD) absolute percentage difference regarding HRs with the same 15 KM survival curve graphs with the preprocessing step was 4.08 % (5.63 %) and the mean (SD) percentage of non-overlapping regions was 5.33 % (5.10 %)). Another thing that can affect the accuracy of IPD is whether users provide numbers at risk or not. There are cases when KM survival curve graphs are not supplemented by numbers at risk. But researchers may have interest in those survival analysis results. However, we should be careful about using reconstructed IPD without numbers at risk as the performance of the algorithms are not reliable in terms of reconstructed HRs and CIs in such cases. We performed a sensitivity analysis by comparing HRs (CIs) reconstructed without providing numbers at risk to the iterative algorithm. The results were based on the same 15 KM survival curve graphs used in the results generated without the preprocessing step. When numbers at risk are not provided (and the total number of patients at baseline are provided), the reconstructed IPD exhibited considerably larger discrepancies from the original data. The mean (SD) absolute HR difference was 6.62 % (6.07 %), and the mean (SD) percentage of non-overlapping region was 21.50 % (22.35 %), which were both larger than the results when numbers at risk were provided. Therefore, we recommend that the reconstruction approach should only be used when numbers at risk are available, to ensure higher fidelity to the original trial data.

As we showed in the Evaluation Section, most of the estimated HRs and their CIs based on the reconstructed IPD are considerably close to those based on the actual data. However, reconstruction accuracy depends heavily on how users precisely digitized KM survival curves. A KM graph with low resolution, curves with extensive censoring symbols or curves of different groups significantly overlapping could lead to major discrepancies between the reconstructed and actual IPD. On the other hand, sparse censoring can enhance the accuracy of IPD reconstruction—since the iterative algorithm distributes censoring times evenly across certain time intervals, ignoring the censoring marks from actual KM survival curve images. As a result, with few censoring marks, the possibility of wrong estimate of event or censoring times get reduced. Another variation may come from using different analysis methods to calculate HR. Some trials computed HRs based on a stratified Cox regression model, as these trials used stratified randomization [[17], [18], [19], [20], [21]]. In our analysis, all reconstructed hazard ratios (HRs) were estimated using Cox regression models that included only a group indicator as the sole covariate. This approach was necessary because the selected published papers generally did not provide stratified survival analyses regarding the stratification variables used. While most of the included RCTs implemented stratification at the randomization stage (e.g., by histological subtype, prior treatment experience, or treatment line), such variables are typically used in statistical models such as stratified Cox regression or stratified log-rank tests. Unless the original publication provides KM survival curves for specific subgroups corresponding to these stratification factors, it is not possible to restore or impute these variables during IPD reconstruction. As such, we caution that reconstructed IPD should not be interpreted as equivalent to the original, actual patient-level data, especially regarding subgroup or stratification analyses when stratification variables are not available. Researchers conducting secondary analyses or meta-analyses using reconstructed IPD without access to stratification variables should do so with care and acknowledge this limitation in their interpretation. In addition, we explored the KM survival curve graphs, numbers at risk, and other milestone survival probabilities. These factors were similar across reconstructed and actual IPD. Thus, it may imply that the effect of stratification variables on the efficacy of a treatment was not considerable in the chosen RCTs.

There are multiple limitations in this study. The evaluations were based on RCTs of one type of treatment (e.g. immuno-oncology). Other treatments for different diseases can have a dissimilar trend in KM survival curves that make digitizing comparatively tricky, resulting in the poor quality of reconstructed IPD. Another constraint is that adaptively preprocessing the KM survival curve graph from digitizing step can't be reproduced since it is manually done, not automatically. In this reason, a direct comparison of multiple reconstruction methods was not feasible for this evaluation. Nevertheless, we utilized the R code provided by Guyot et al. [10] to quantify the performance of the original method. As this original algorithm does not include preprocessing features, we manually prepared the digitized coordinates to meet survival curve properties (i.e., ensuring the initial point is (0, 1) and correcting any non-monotonic survival probability regions). These steps were necessary to ensure algorithmic convergence and the validity of the reconstructed data. The mean (SD) absolute percentage difference in reconstructed HRs compared to published values was 2.93 % (3.34 %), and the mean (SD) percentage of non-overlapping regions for CIs was 5.74 % (4.92 %). While both methods performed comparably, the Liu's implementation [12] resulted in slightly lower bias and improved agreement in CI overlap. Furthermore, we found the method by Liu et al. [12] to be more user-friendly, as it provides an integrated preprocessing step for ensuring valid and monotonic survival curves prior to reconstruction. In addition, no further examination was done regarding factors (e.g. sample size, follow-up length, etc) that might affect the differences between reconstructed and actual IPD. To empirically see any pattern in the performance with different sample sizes or follow-up lengths, we recorded those data (sample size and follow-up length) of the chosen 46 KM survival curve graphs. Our findings indicate that smaller sample sizes (e.g., 33, 36, 47, and 92) were associated with greater discrepancies between original and reconstructed HRs, with absolute percentage differences of 4.14 %, 5.68 %, 5.71 %, and 4.62 %, respectively. However, as no formal simulation study was performed, this finding should not be generalized. Regarding follow-up length and at-risk numbers presented in the original papers, we did not observe a consistent pattern in HR differences or non-overlapping CI regions. Thus, further simulation studies are warranted to more thoroughly investigate these effects.

Another limitation is that all reconstructions of the 46 Kaplan-Meier survival curve graph were performed by a single analyst. This approach was chosen primarily due to practical constraints, including the time-intensive and laborious nature of the digitization process, as well as the learning curve required for consistent and accurate extraction. We acknowledge that having multiple independent reviewers could improve the reliability and objectivity of the digitized data by enabling assessment of inter-rater variability. To mitigate potential subjectivity, the analyst involved in this study underwent extensive practice digitizing KM survival curves prior to reconstructing IPD from the selected RCTs. In future applications, we recommend that, when feasible, digitization be performed independently by multiple analysts. Their extracted results can then be compared to identify regions of discrepancy. Then, extracted coordinates from those regions can be modified by checking the reason for the discrepancy, thereby improving overall accuracy and reproducibility.

Finally, we would like to emphasize that reconstructed IPD are inherently approximate. We caution that secondary, or meta-analyses relying on reconstructed IPD without access to original patient-level data or stratification variables, the results should be interpreted with appropriate care. Nonetheless, reconstructed IPD can be valuable in methodological research, for comparative studies of statistical methods, and as supplementary data for grant applications or exploratory work—offering a more realistic benchmark than simulated survival data under idealized conditions.

Through the extensive application of the reconstruction methods, we were able to easily reconstruct IPD from multiple RCTs and get the parameter estimates of the efficacy of treatment close to those reported in published papers. These reconstructed IPD could be useful for designing future clinical trials or conducting secondary data analyses.

## CRediT authorship contribution statement

**Jaromme Kim:** Validation, Formal analysis, Writing – original draft, Methodology, Data curation, Visualization, Investigation. **Prabhakar Chalise:** Supervision, Writing – review & editing, Validation. **Jianghua He:** Validation, Conceptualization, Supervision, Writing – review & editing, Methodology.

## Declaration of generative AI and AI-assisted technologies in the writing process

During the preparation of this work the author(s) used Grammarly in order to check spelling errors. After using this tool/service, the author(s) reviewed and edited the content as needed and take(s) full responsibility for the content of the publication.

## Fund

This research was supported in part by the National Cancer Institute Cancer Center Support Grant P30 CA168524 and Department of Biostatistics and Data Science at University of Kansas Medical Center.

## Declaration of competing interest

The authors declare that they have no known competing financial interests or personal relationships that could have appeared to influence the work reported in this paper.
