# Supplementary material for: Reconstructing patient level survival data from published Kaplan-Meier curves
Source: Contemp Clin Trials Commun. 2025 Aug 20;47:101542. doi: 10.1016/j.conctc.2025.101542 (PMC12409465; doi:10.1016/j.conctc.2025.101542)
Supplement: Multimedia component 1 [file mmc1.pdf]

Number	First Author & Type of Graph	HR original (CI)	HR reconstructed (CI)
1	Borghaei OS	0.73 (0.59, 0.89)	0.761 (0.62, 0.93)
2	Borghaei PFS	0.92 (0.77, 1.11)	0.901 (0.75, 1.08)
3	Fehrenbacher High Gene Subgroup	0.43 (0.24, 0.77)	0.53 (0.31, 0.90)
4	Fehrenbacher Low Gene Subgroup	1.1 (0.68, 1.76)	1.03 (0.66, 1.59)
5	Fehrenbacher ITT	0.73 (0.53, 0.99)	0.73 (0.54, 0.98)
6	Fehrenbacher TC0 and IC0	1.04 (0.62, 1.75)	1.09 (0.64, 1.86)
7	Fehrenbacher TC1/2/3 or IC1/2/3	0.59 (0.4, 0.85)	0.60 (0.41, 0.87)
8	Fehrenbacher TC2/3 or IC2/3	0.54 (0.33, 0.89)	0.55 (0.34, 0.91)
9	Fehrenbacher TC3 or IC3	0.49 (0.22, 1.07)	0.46 (0.21, 1.01)
10	Herbst Any PD-L1	0.83 (0.65, 1.07)	0.85 (0.66, 1.08)
11	Herbst High/Intermediate PD-L1	0.72 (0.52, 0.99)	0.73 (0.52, 1.02)
12	Herbst High PD-L1 on 22C3 Assay	0.6 (0.42, 0.86)	0.61 (0.42, 0.87)
13	Herbst High PD-L1 on SP263 Assay	0.71 (0.5, 1.0)	0.71 (0.50, 1.01)
14	Herbst High PD-L1	0.59 (0.4, 0.89)	0.61 (0.40, 0.91)
15	Kantoff Primary Efficacy	0.78 (0.61, 0.98)	0.76 (0.60, 0.95)
16	Larkin ITT - Nivolumab plus versus Ipilimumab	0.42 (0.31, 0.57)	0.44 (0.33, 0.60)
17	Larkin ITT - Nivolumab versus Ipilimumab	0.57 (0.43, 0.76)	0.62 (0.46, 0.83)
18	Larkin ITT - Nivolumab plus versus Nivolumab	0.74 (0.6, 0.92)	0.77 (0.62, 0.97)
19	Mok EGFR-Mutation Negative	2.85 (2.05, 3.98)	2.84 (2.05, 3.94)
20	Mok EGFR-Mutation Positive	0.48 (0.36, 0.64)	0.49 (0.37, 0.64)
21	Mok Unknown EGFR-Mutation Status	0.68 (0.58, 0.81)	0.66 (0.56, 0.77)
22	Mok OS	0.74 (0.65, 0.85)	0.74 (0.65, 0.85)
23	Motzer PD-L1 ≥ 1%	0.79 (0.53, 1.17)	0.79 (0.53, 1.17)
24	Motzer PD-L1 < 1%	0.77 (0.6, 0.97)	0.77 (0.61, 0.98)
25	Motzer OS	0.73 (0.57, 0.93)	0.77 (0.63, 0.94)
26	Motzer PFS	0.88 (0.75, 1.03)	0.87 (0.74, 1.01)
27	Perris PD-L1 ≥ 1%	0.55 (0.36, 0.83)	0.55 (0.37, 0.83)
28	Perris PD-L1 < 1%	0.89 (0.54, 1.45)	0.87 (0.53, 1.41)
29	Perris OS	0.7 (0.51, 0.96)	0.67 (0.49, 0.92)
30	Perris PFS	0.89 (0.7, 1.13)	0.91 (0.71, 1.16)
31	Postow PFS	0.4 (0.23, 0.68)	0.40 (0.23, 0.69)
32	Rizvi OS - Durvalumab versus Chemo	0.76 (0.56, 1.02)	0.74 (0.55, 0.99)
33	Rizvi OS – Durvalumab + versus Chemo	0.85 (0.61, 1.17)	0.84 (0.61, 1.16)
34	Rizvi OS bTMB ≥20 mut/Mb – Durvalumab + versus Durvalumab -	0.74 (0.48, 1.11)	0.74 (0.49, 1.14)
35	Rizvi OS bTMB ≥20 mut/Mb – Durvalumab versus Chemo	0.72 (0.5, 1.05)	0.70 (0.48, 1.04)
36	Rizvi OS bTMB ≥20 mut/Mb – Durvalumab + versus Chemo	0.49 (0.32, 0.74)	0.51 (0.34, 0.76)
37	Rizvi OS bTMB < 20 mut/Mb – Durvalumab + versus Durvalumab	1.22 (0.98, 1.52)	1.21 (0.86, 1.70)
38	Rizvi OS bTMB < 20 mut/Mb – Durvalumab versus Chemo	0.93 (0.74, 1.16)	0.93 (0.74, 1.16)
39	Rizvi OS bTMB < 20 mut/Mb – Durvalumab + versus Chemo	1.16 (0.93, 1.45)	1.16 (0.93, 1.45)
40	Rizvi PFS – Durvalumab versus Chemo	0.87 (0.59, 1.29)	0.89 (0.61, 1.31)
41	Rizvi PFS – Durvalumab + versus Chemo	1.05 (0.72, 1.53)	1.08 (0.74, 1.57)
42	Rizvi PFS bTMB ≥20 mut/Mb - Durvalumab + versus Durvalumab	0.76 (0.5, 1.15)	0.73 (0.48, 1.12)
43	Rizvi PFS bTMB ≥20 mut/Mb - Durvalumab versus Chemo	0.77 (0.52, 1.13)	0.75 (0.51, 1.09)
44	Rizvi PFS bTMB ≥20 mut/Mb - Durvalumab + versus Chemo	0.53 (0.34, 0.81)	0.49 (0.32, 0.75)
45	Rizvi PFS bTMB < 20 mut/Mb - Durvalumab + versus Durvalumab	1.26 (1.02, 1.57)	1.28 (1.03, 1.60)
46	Rizvi PFS bTMB < 20 mut/Mb - Durvalumab versus Chemo	1.19 (0.94, 1.5)	1.19 (0.94, 1.50)
47	Rizvi PFS bTMB < 20 mut/Mb - Durvalumab+ versus Chemo	1.55 (1.23, 1.94)	1.57 (1.25, 1.97)
48	Robert OS	0.42 (0.25, 0.73)	0.43 (0.25, 0.73)
49	Robert PFS	0.43 (0.34, 0.56)	0.46 (0.36, 0.59)
50	Shitara OS PD-L1 CPS ≥1	0.91 (0.69, 1.18)	0.89 (0.68, 1.15)

51	Shitara OS PD-L1 CPS ≥10	0.69 (0.49, 0.97)	0.67 (0.48, 0.94)
52	Shitara OS PD-L1 CPS ≥1 – Pembrolizumab + versus Chemo	0.85 (0.7, 1.03)	0.83 (0.69, 1.01)
53	Shitara OS PD-L1 CPS ≥10 – Pembrolizumab + versus Chemo	0.85 (0.62, 1.17)	0.85 (0.62, 1.17)
54	Shitara MSI-H Tumors – Pembrolizumab versus Chemo	0.29 (0.11, 0.81)	0.30 (0.11, 0.84)
55	Shitara MSI-H Tumors – Pembrolizumab+ versus Chemo	0.37 (0.14, 0.97)	0.39 (0.15, 1.03)
56	Wolchok OS - 10mg/kg versus 0.3mg/kg	0.77 (0.53, 1.13)	0.76 (0.52, 1.12)
57	Wolchok OS – 10mg/kg versus 3.0mg/kg	0.88 (0.59, 1.29)	0.88 (0.59, 1.30)
58	Wolchok OS – 3.0mg/kg versus 0.3mg/kg	0.88 (0.60, 1.28)	0.88 (0.60, 1.28)
